# Autistic-Delivered Peer Support: A Feasibility Study

**DOI:** 10.1007/s10803-022-05816-4

**Published:** 2022-11-12

**Authors:** Lindsay L. Shea, Mi-Yeet Wong, Wei Song, Katy Kaplan, Disha Uppal, Mark S. Salzer

**Affiliations:** 1 A.J. Drexel Autism Institute, Drexel University, 3020 Market Street, Suite 560, Philadelphia, PA 19104 USA; 2 College of Public Health, Temple University, 1101 W Montgomery Avenue, Philadelphia, PA 19122 USA; 3 Community Behavioral Health, 801 Market St, Philadelphia, PA 19107 USA

**Keywords:** Autism, Peer support, Peer, Peer specialist, Autistic, Service delivery

## Abstract

Peer support has been an undeveloped pathway for filling the service gap and to generate employment opportunities for autistic individuals. Peer supports have been deployed widely in mental health and among veterans and understanding the utility of this service modality among autistic individuals illuminates opportunities for research, policy, and practice. This study examined characteristics of participants in an autistic-delivered peer support program and reports on use of and satisfaction with the program. Half of autistic participants had a co-occurring mental health diagnosis. Participants reported multiple areas of unmet needs and participant satisfaction with the program was high (90%). The findings of this study point toward autistic-delivered peer support as a promising avenue for future development.

**Supplementary Information:**

The online version contains supplementary material available at 10.1007/s10803-022-05816-4.

A robust and growing evidence base has identified gaps in services and supports available to autistic adolescents and adults (Dudley et al., 2019; Kałużna-Czaplińska et al., 2018; Koffer Miller et al., 2018; Myers et al., 2015; Turcotte et al., 2016). The creation of services to meet the needs of this group is urgent, as the number of autistic youth aging into adulthood is growing (Schott et al., 2021a, 2021b; Shattuck et al., 2012). As autistic youth age, poor outcomes have been observed in multiple areas, including social and community participation, employment, interpersonal relationships, independent living, and overall quality of life (Henninger & Taylor, 2013; Roux et al., 2015; Shattuck et al., 2011, 2012; Sosnowy et al., 2018; Wehman et al., 2014). A recent meta-analysis found that approximately 50% of the autistic adults included in research studies did not achieve independence in those areas (Mason et al., 2021). Unmet needs for services to support social skills, adaptive skills such as self-care, self-acceptance, and other skill areas may result in barriers for autistic individuals to live independently and fully participate in their community (Cage et al., 2017; Henninger & Taylor, 2013; Kanne et al., 2011). Generating new service options to meet the needs of autistic adolescents and adults that can be funded by Medical Assistance, or Medicaid, is especially critical since it is a primary insurer relied upon by this group across the lifespan (Rizzolo et al., 2013; Schott et al., 2021a, 2021b; Semansky et al., 2011b).

Peer support services delivered by autistic individuals are a potentially promising avenue for addressing varying support needs of autistic individuals in many areas, such as community participation, employment, skill building, and social relationships. Such services are widely established across mental health systems in the United States (Mental Health America, 2021; Open Minds, 2018) and a fundamental service of Centers for Independent Living, which are federally funded programs that offer community living supports for all individuals with disabilities (Salzer et al., 2016; White et al., 2010). The lived experiences of autistic people underlie their appreciation of the importance of self-determined participation in different life domains. Moreover, the likely effectiveness of autistic-delivered peer support is based on robust theories, including social learning theory, social comparison theory, the value of experiential knowledge, and the helper-therapy principle (Salzer, 2002).

Autistic peers offer novel opportunities to enhance independent living and participation of autistic individuals. Empirical studies have demonstrated that autistic peers have fewer stigmatizing beliefs and attitudes toward autism than non-autistic people (Bertilsdotter-Rosqvist, 2019; Gillespie-Lynch et al., 2017). Moreover, recent research has indicated that autistic adults also prefer to interact with autistic adults over non-autistic adults and can effectively communicate information to their autistic peers (Crompton et al., 2020; Morrison et al., 2020). Autistic people can develop close bonds, demonstrate empathy, and have less stressful communication with autistic people than with non-autistic people (Crompton et al., 2020; Morrison et al., 2020). These findings indicate that autistic-led services may be particularly beneficial to autistic individuals. The rise in autistic self-advocacy and the neurodiversity movement also calls for the development of interventions that support autistic people to determine outcomes that are relevant to their quality of life instead of measures based on typical neurodevelopment (Pellicano & Houting, 2022). In alignment with the neurodiversity paradigm, peer-led services aim to help autistic people overcome challenges and lead meaningful lives while valuing all people for who they are and as they are.

Systematic literature reviews have found that most peer support programs involving individuals on the autism spectrum have focused on children and have occurred in educational settings (Chan et al., 2009; Chang & Locke, 2016). Who constitutes a “peer” is often different in autism peer support interventions compared to interventions in other disability populations. In published research involving other disability populations, peer support is commonly delivered by individuals who experience similar issues or conditions (Lloyd-Evans et al., 2014; Shalaby & Agyapong, 2020). In contrast, scholarly peer support research involving autistic individuals predominantly involves peers who are typically developing youth of the same age who have been nominated by their teachers and/or other students, rather than autistic individuals (Corbett et al., 2014; Kamps et al., 2015; Kasari et al., 2012; Roeyers, 1996). The most common components of these programs were having the similarly aged peer initiate interactions with the autistic participant and maintain a positive relationship, prompt or support participants to engage in various behaviors, provide contingent reinforcement, and offer academic instruction (Chan et al., 2009).

The limited research on peer support among autistic adults has primarily examined interventions in postsecondary university settings. A recent systematic review identified nine adult ASD peer support programs in these settings that reported positive outcomes in social skills, academic performance, and sense of belonging (Duerksen et al., 2021). Similar to studies focused on children and adolescents, most of these peer support programs involved typically developing peers who received specialized training to work on participant goals in both one-to-one and group interactions (Ames et al., 2015; Duerksen et al., 2021; Roberts & Birmingham, 2017; Siew et al., 2017; Todd et al., 2019).

Among the few autistic-delivered peer support programs that have been described in the literature, one Swedish program focuses on life strategies (Rosqvist, 2019). A group of autistic self-advocates are engaged in teaching, course development, handling practical matters, managing contacts with social services, performing employer-related group leadership functions, or administrative work. Focus group findings with program members suggest that supports offered by peers on the spectrum emphasize their strengths whereas non-autistic delivered supports tend to focus on hardships and difficulties with social interactions. Non-autistic supporters also focused on autistic challenges, provided more support than the participants reported was needed, and sometimes involved supporters who spoke on behalf of autistic people rather than supporting people to speak for themselves. Autistic participants who were supported by non-autistic “peers” often described feeling misunderstood which prevented them from taking part in experiences that would encourage their problem-solving abilities (Rosqvist, 2019).

The relative dearth of research on autistic-delivered peer support may be due to a variety of issues. First, there may be concerns about the value of peer support services delivered by individuals on the autism spectrum to others on the autism spectrum. Research has suggested that some providers have historically expressed concerns about peer support services delivered by individuals with mental health diagnoses to others with mental health diagnoses because they viewed them as potentially being unhelpful, at best, and harmful, at worst (Lee, 1995). One study found that mental health professionals viewed peer-based interventions as less helpful than professionally-delivered services, despite the lack of evidence to make such a judgment (Salzer et al., 1994). Other research suggests that while many mental health professionals have some degree of comfort with peer support offered by individuals with mental health diagnoses, 16% would try to dissuade individuals from obtaining such support in favor of services delivered by non-peer professionals and 37% would not be in favor of funding such initiatives if they competed for funding with non-peer professional services (Salzer et al., 2001). Peers with mental health issues are now a growing part of the mental health workforce (Salzer, 2010), but the extent to which this has altered professional views of the value of peer support services remains unclear. Similar research pertaining to traditional provider beliefs about autistic-delivered peer support does not exist. There is research, however, showing that approximately two-thirds of parents of autistic children have participated in support groups that involve some degree of parent-to-parent support (Mandell & Salzer, 2007), but provider perspectives about these groups have not been documented. A second possible barrier to the emergence of autistic-delivered peer support may be concerned about the development of positive interpersonal bonds with their peers, which is important for positive peer support outcomes (Thomas & Salzer, 2018).

The goal of this paper is to examine areas of feasibility (i.e., demand, practicality, and acceptability; Bowen et al., 2009) of an autistic-delivered peer support program aimed at enhancing self-identified goals for community outcomes among autistic adolescents and adults. The overall goal of the initiative, the Community Autism Peer Specialist (CAPS), is to promote independent living, participation, and social relationships of autistic youth and adults by addressing individual-identified needs and goals. The CAPS initiative involved the development of a first of its kind peer specialist training program designed by and for autistic peers, which we describe briefly, followed by a description of the CAPS program itself that employed some of the trained peer specialists. We examine the level of utilization of the CAPS services and characteristics of those who have participated in the CAPS program, such as age, co-occurring conditions, social functioning, and unmet needs, with a particular interest in assessing whether autistic individuals with more service needs are willing to seek autistic-delivered peer support services. We also demonstrate practicality through self-reported relationships between peer specialists and program participants in agreement on the goals and tasks and development of bond. Moreover, we evaluate acceptability by assessing both participants and peer specialists’ satisfaction with the program. This information is critical to informing future efforts aimed at expanding the availability of similar peer support initiatives for autistic individuals focused on enhancing community outcomes.

## Development of the CAPS Program

### Creation of the CAPS Training Curriculum

Medicaid-reimbursed peer support services delivered by individuals with mental health diagnoses to others with mental health diagnoses require peer specialists to receive specialized peer support training (Salzer, 2010). Training programs involving typically 40–80 hours of didactic, role play, and other activities were initially established in 2004 in the mental health arena across the United States and have been shown to be effective in enhancing knowledge and positive employment outcomes (Salzer, 2010). These training programs lead to a certification that is often referred to as “Certified Peer Specialist” which makes them eligible to deliver Medicaid billable services. This process is being increasingly overseen by state-run Certification Boards across the United States.

The training curriculum was facilitated by a consultant group comprised of neurodiverse individuals to ensure that culture, the nuance of language, and content were reflective of autistic individuals’ perspectives. Specifically, the consultant group led the process of seeking feedback from an Advisory Board and Curriculum Committee on the development of the training curriculum materials and content formatting (e.g., layout, table of content). Committee members completed a survey that asked whether certain content should be included or not and changes that they would recommend. Furthermore, the peer support curriculum used in the mental health field was compared to areas where autism-specific content would need to be newly created or modified from a mental health-specific lens.

The intention of the CAPS program was to structure training and service delivery to be positioned to obtain approval as a Medicaid billable service similar to what is required for billing for peer support for individuals with mental health diagnoses. In parallel to the required length of typical Certified Peer Specialist training in mental health in Pennsylvania (Pennsylvania Certification Board, 2020), the CAPS training program was developed to be 75 hours long. Specifically, the training involved 12, 4–8 hour, in-person sessions delivered over the span of four weeks. The curriculum focused on eight areas: building self-knowledge, advocacy & goals, school life, relationships, home life, community & services, working & volunteering, and life after completing the training. There were four trainers including an autistic individual who was also a certified peer specialist, a parent of an autistic youth who was also a certified peer specialist, a trainer for a peer support provider, and staff from a local university autism center. The sessions included time allotted for the CAPS to job shadow another peer specialist in the field. To graduate with a certificate of completion, CAPS trainees were expected to attend all sessions, complete all assignments, and be active participants in the program.

### CAPS Program and Its Conceptual Model

**Fig. 1:**
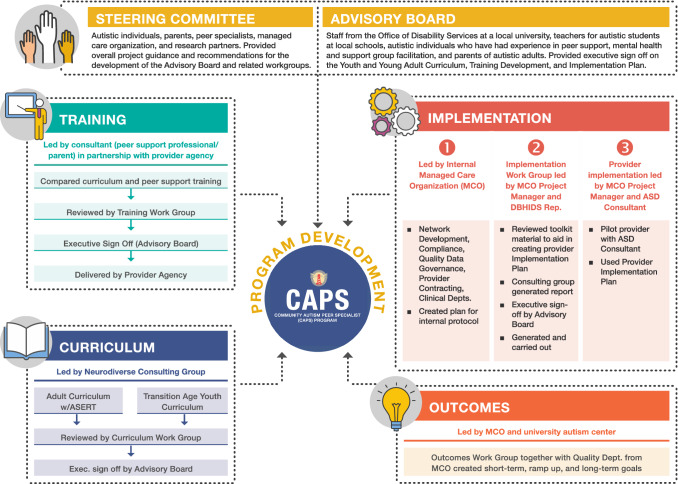
CAPS Development Process

The CAPS program was created in a large, diverse city in the fifth most populous state in the US (Pennsylvania State Data Center Penn State Harrisburg, 2021; see Fig. 1 for the process). A Steering Committee guided the implementation of the CAPS program, including the training development, consisted of smaller workgroups focused on program components (curriculum development and training implementation), service delivery (job descriptions, provider supports and resources, and funding sustainability), and evaluating outcomes. The Steering Committee was comprised of 22 members who convened over the course of 7 meetings. Members represented the Medicaid managed care organization’s executive sponsorship and provider network management, certified peer specialists, autism program administrators, mental health and peer support provider agency where the program would be implemented, autistic individuals and family members with lived and professional experience in the peer support field, and a university autism center.

An Advisory Board consisting of a diverse group of 10 members including autistic individuals, family members, service providers, and policymakers provided guidance on the curriculum materials, training, and the CAPS implementation process. The Advisory Board met monthly during CAPS startup and included autistic individuals with experience in peer support, staff from the Office of Disability Services at a local university, autism support classroom teachers from local schools, mental health and support group facilitators, and parents of autistic adults. Ensuring that autistic voices and individuals with lived experience were embedded in the program development was a top priority, as it led to formative discussions on the purpose and philosophy of CAPS.

**Fig. 2:**
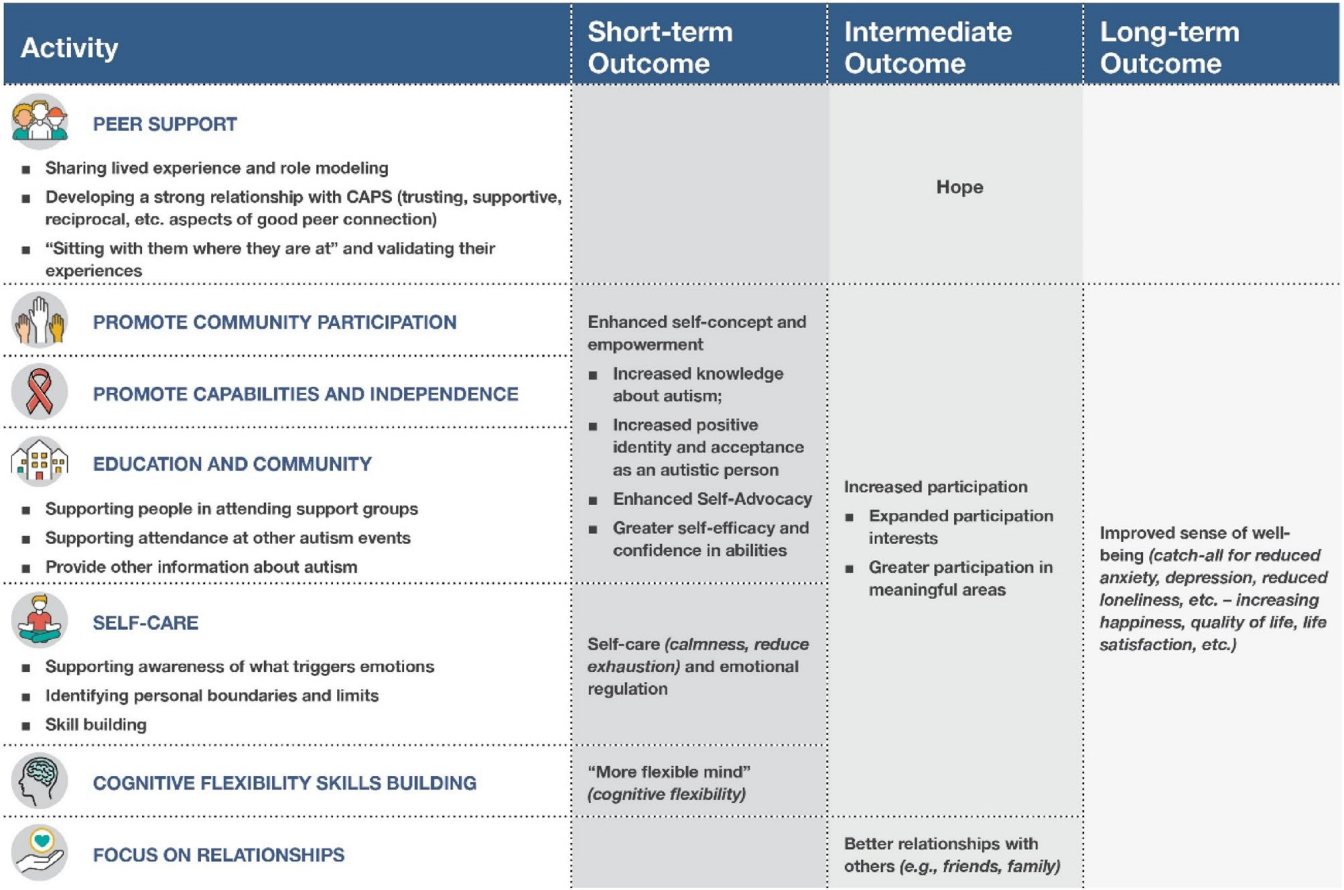
CAPS Conceptual Model

The conceptual framework underlying CAPS resulted from an intensive series of meetings with the CAPS Director, CAPS Program Supervisor, and six CAPS peer specialists, with particular attention paid to articulating CAPS activities and their associated outcomes. The CAPS Director oversaw the implementation of the program at the local mental health peer support provider agency and communication with the Medicaid managed care organization. The CAPS Program Supervisor provided oversight and supervision to the Peer Specialists. The six CAPS Peer Specialists provided the CAPS service to program participants. The series of meetings on the conceptual framework identified seven primary activities that the CAPS Peer Specialists worked with participants on: (1) providing peer support (sharing lived experiences, developing relationships, validating experiences), (2) promoting community participation, (3) promoting capabilities and independence, (4) supporting education on autism characteristics (attending support groups, autism events, increasing awareness of autism), (5) awareness and practice of self-care, (6) building cognitive flexibility skills, and (7) focusing on relationships. The short-term, intermediate, and long-term outcomes associated with each activity were also identified, including hope, enhanced self-concept and empowerment, increased participation, better relationships, and improved sense of well-being (Fig. 2).

## Methods

### Procedures

#### Implementation of the CAPS Program

CAPS primarily involved one-to-one, face-to-face interactions between program participants and a peer specialist with occasional group events. The COVID pandemic in 2020 necessitated a transition to virtual interactions for various periods of time. The CAPS Peer Specialists worked in partnership with participants to identify their goals in the domains of living, education, working, wellness, and social domains, as well as the skills, supports, and resources needed to accomplish the goals. They then worked together to complete a goal plan which identifies the participant’s needs and action steps to achieve identified goals. The peer specialist and participant worked together to determine the frequency and duration of their meetings, but they typically met at least weekly.

Service providers that service autistic individuals were made aware of the availability of CAPS through direct outreach to generate referrals for the CAPS service. Stakeholders in the development of the CAPS initiative also promoted the program through webinars to the public and sessions at autism and peer specialist-related conferences. Inclusion criteria for receiving the CAPS service included being 14 or older with an autism spectrum disorder (ASD) diagnosis and being eligible for Medicaid. Individuals were enrolled through referrals by licensed treatment providers. After the referral, interested participants completed an intake evaluation form. This was reviewed by the CAPS program leadership who matched the individual to a peer specialist based on their availability, shared interests, and preferences of the individual, including gender preferences (for example, if a participant prefers a female CAPS), location (office, community, or home visits), and transportation use to ease travel for the participants and CAPS.

### Data Collection

A set of measures were established by members of the outcomes evaluation subcommittee of the Steering Committee. Measures were compiled into an online survey tool (REDCap) (Harris et al., 2009), pilot-tested among the subcommittee members, and finally used with CAPS peer specialists and participants. A completed baseline measure, which included demographic and clinical items, was required prior to receiving CAPS services. The online survey link was distributed to CAPS peer specialists and participants by the agency overseeing service delivery at baseline and at 3-month intervals. The baseline and the first 3-month post-baseline assessment are the focus of this feasibility study. Data was collected from September 2019 to January 2021. The City of Philadelphia Department of Public Health Institutional Review Board approved this study (Assurance Identification No. FWA00003616).

### Participants

### CAPS Participants

A total of 29 CAPS participants were enrolled at the time this study was conducted. Most were male (n = 23, 79%), and the average age was 19.7 (SD = 6.1), with a range of 14–41. Eleven participants were 19 years old and older. Most participants identified as white (44.8%) or Black (41.1%), and 13.8% were Hispanic (Table 1). Fewer than one in ten participants were employed (n = 2, 6.9%). Regarding their co-occurring conditions, almost half of participants (n = 14) indicated co-occurring mental health diagnoses of anxiety, depression, attention deficit hyperactivity disorder (ADHD), bipolar, or posttraumatic stress disorder. This percentage was lower than the prevalence of co-occurring mental health conditions in the autistic adult sample in previous studies (Buck et al., 2014; Lever & Geurts, 2016). Specifically, 31.0% of participants (n = 9) reported anxiety, followed by 27.6% (n = 8) having attention deficit hyperactivity disorder (ADHD) and 20.7% (n = 6) having depression. Only one participant reported a diagnosis of intellectual disability.

**Table 1:** Baseline demographic characteristics of Participants Receiving the Community Autism Specialist (CAPS) Service (n = 29)

	Program participants
	*n (%)*
Gender	
Male	23 (79.3)
Female	5 (17.2)
Other	1 (3.4)
Race and Ethnicity	
Black	12 (41.1)
White	13 (44.8)
Hispanic	4 (13.8)
Age	
Average Age (SD)	19.7 (6.1)
Age Range	14 to 41
Intellectual Disability	1 (3.4)
Mental health Diagnoses	
Attention Deficit Hyperactivity Disorder	8 (27.6)
Depression	6 (20.7)
Anxiety	9 (31)
Posttraumatic Stress Disorder	2 (6.9)
Bipolar	2 (6.9)
Any mental health diagnosis	14 (48.3)
Raw SRS-2 Score	95.9 (23.4)
SRS-2 Category	
Normal range	2 (7.1)
Mild range	7 (25.0)
Moderate range	13 (46.4)
Severe range	6 (21.4)
Employed	2 (6.9)
Average Number of Desired Needs	9.4 (4.3)
Number of peers who visited an emergency room, hospital, or crisis center for mental health, psychiatric, or emotional help at any point in their life	9 (31.0)
Average number of visits to an emergency room, hospital, or crisis center for mental health, psychiatric, or emotional help at any point in their life	1.3 (2.9)
Number of peers who visited an emergency room, hospital, or crisis center for mental health, psychiatric, or emotional help in the last 12 months	4 (13.8)
Average number of visits to an emergency room, hospital, or crisis center for mental health, psychiatric, or emotional help in the past 12 months	0.4 (1.3)

#### Peer Specialists

In order to receive the CAPS training, peer specialists must be: (1) being 18 years of age or older with an autism diagnosis, (2) having a high school diploma or GED from an accredited institution, and (3) having engaged in employment or education within the past three years (maintained at least 12 months of successful full, part-time, or voluntary work experience, or obtained at least 24 credit hours of post-secondary education). A total of six peer specialists were employed to provide CAPS services during the course of this study. The average age of the peer specialists was 29.7 (SD = 28, ranging from 21 to 46) and four (66.7%) were female. Three were Non-Hispanic white, one Black, one Native Hawaiian or Other Pacific Islander, and one other race/ethnicity. Four out of the six peer specialist had at least one mental health diagnosis. Specifically, two peer specialists had ADHD, two had depression, and two had anxiety. No peer specialists in this study reported an intellectual disability diagnosis.

### Measures

The following measures were used to assess feasibility of the CAPS program. First, to assess the demand of the program, we used demographic and clinical information, Social Responsiveness Scale 2nd edition (SRS-2), and Camberwell Assessment of Needs to examine the characteristics of those who participated in the program. We also used utilization of the CAPS services to assess the demand. Second, to assess the practicality, we used the Working Alliance Inventory-Short Form to measure the level of agreement on the goals and tasks and development of bond. Third, to measure the acceptability, we asked participants and peer specialists to rate their satisfaction with the program.

#### Self-Reported Socio-Demographic and Clinical Information (Baseline)

Self-reported gender, race and ethnicity, and age were collected at baseline and co-occurring psychiatric diagnoses collected included diagnoses of Attention Deficit Hyperactivity disorder (ADHD), Depression, Anxiety, Post Traumatic Stress disorder (PTSD), Bipolar disorder, or Intellectual Disability (ID). Crisis service use history was captured by asking participants to enter the number of times they had gone to an emergency room, hospital, or crisis center for mental health, psychiatric or emotional help in their lifetime and within the last 12 months. Employment status was identified by asking whether participants had a job where they were earning at least minimum wage.

#### ASD Traits (baseline)

The SRS-2 Adult Self Report was used at the baseline to measure the ASD traits. This 65-item instrument was scored on a 4-point Likert scale, from Not True to Almost Always True. The total score measures social deficits, with T-scores of $\ge$ 76 considered severe, between 66 and 75 considered moderate, between 60 and 65 considered mild, and $\le$ 59 or below considered normal (Bruni, 2014).

#### Needs Assessment (baseline)

A modified version of the Camberwell Assessment of Needs (Phelan et al., 1995) was used to identify self-reported care needs of CAPS participants. It assesses unmet needs in life domains and if help within that domain is a goal within the next year. This assessment has demonstrated inter-rater (r = 0.99) and test–retest reliability (r = 0.78) among people with severe mental health diagnoses (Phelan et al., 1995). There are 23 life domains, including housing, food, daytime activities, mental health care needs, autism information and treatment, social life, education, and employment. A desired need in a particular area was identified when a participant indicated that they had a need in the area and desired support to address that need. The total number of desired needs was calculated for each individual.

#### Service Utilization (3-month)

Participants self-reported the number of hours they met with a CAPS peer specialist in person in a typical week and the number of times they met with a CAPS peer specialist at the provider location, in the participants’ home, and in the community. Documentation of contacts with CAPS continued with the transition to virtual contacts during the COVID-19 pandemic.

#### Working Alliance Inventory (3-month)

The WAI-S (Tracey & Kokotovic, 1989) was adapted to measure participants’ perceptions of their working relationship with the peer specialist. A total of 12 items (e.g., “I believe my CAPS Peer Specialist likes me,” “My CAPS Peer Specialist and I collaborate on setting goals for my wellness”) were rated on a 1 (seldom) to 5 (always), with higher total scores indicating a greater working alliance (see the full list of adapted items in Appendix A). This 12-item questionnaire had three central dimensions: (a) agreement on the goals (the goal dimension); (b) agreement on tasks to be accomplished to achieve these goals (the task dimension); and (c) the development of a personal bond between the CAPS participants and peer specialists (the bonding dimension). The WAI-S demonstrates acceptable psychometric properties (Busseri & Tyler, 2003).

#### Satisfaction (3-month)

**Fig. 3:**
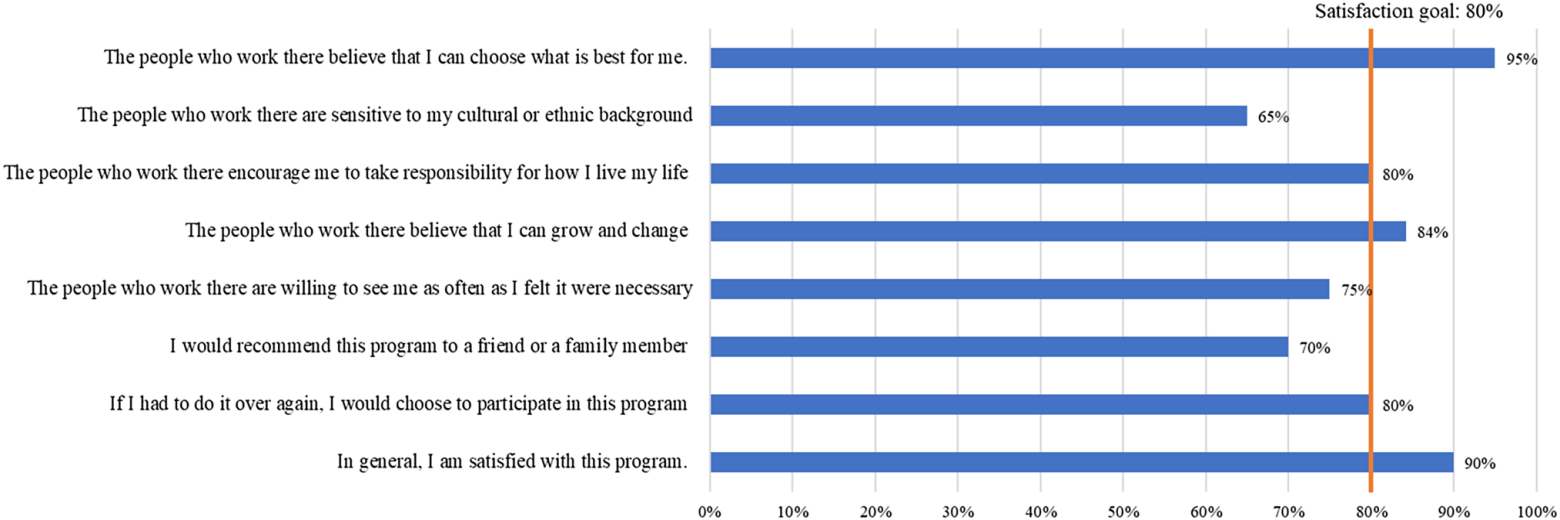
Program satisfaction of CAPS participants after 3-month (N = 20)

**Fig. 4:**
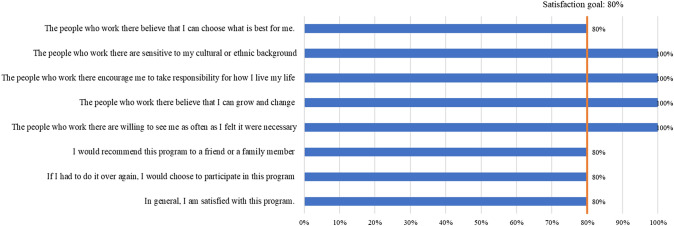
Program satisfaction of CAPS peer specialists after 3-month (N = 5)

Both CAPS participants and peer specialists were asked to respond to a series of eight questions (see Figs. 3 and 4 for the items) about CAPS participation and experiences using a 5-point Likert scale from Strongly Agree to Strongly Disagree. Some example items included “The people who work there believe that I can choose what is best for me,” and “The people who work there are sensitive to my cultural or ethnic background.” Acceptability criteria were set at 80% (4/5) of participants agreed or strongly agreed with the statements. Satisfaction below this proportion was identified as an area for needed programmatic improvement.

## Results

### Baseline Clinical Characteristics

One in three participants (31%) reported going to an emergency room, hospital, or crisis center for mental health, psychiatric or emotional help in their lifetime, with an average of 2.9 visits over their lifetime. Approximately 14% of participants had gone to an emergency room, hospital, or crisis center for mental health, psychiatric or emotional help at least once in the past year with an average of 1.3 visits.

### Baseline ASD Traits

Among the 28 CAPS participants who responded to the SRS-2, six (21.4%) were in the Severe category of social impairment, 13 (46.4%) in the Moderate category, seven (25.0%) in the Mild category, and two (7.1%) in the Normal category.

### Baseline Needs Assessments

On average, participants indicated having a need in 9.4 out of the 23 domains (Table 2). The most frequent need reported was their social life, which was endorsed by 86% of participants. The next most frequently endorsed domains were physical activity and exercise (69%), daytime activities and psychological distress (62%), information about autism and transportation needs (59%), financial needs (55%), and education (52%). The least often reported domains were physical health care needs (3%), religious or spiritual life (7%), and housing and food (both 10%).

**Table 2:** Desired needs of participants receiving CAPS service at baseline

	Participants Receiving CAPS (n = 29) *n (%)*
Social life	25 (86.2)
Physical activity and exercise	20 (69.0)
Daytime activities	18 (62.1)
Psychological distress	18 (62.1)
Information about autism spectrum disorder and treatment	17 (58.6)
Transportation needs	17 (58.6)
Financial needs	16 (55.2)
Education	15 (51.7)
Intimate relationships	14 (48.3)
Legal rights and advocacy	14 (48.3)
Telephone communication	13 (44.8)
Benefits and entitlements	13 (44.8)
Keeping clean and well groomed	12 (41.4)
Relationship with parents and/or siblings	11 (37.9)
Voting, volunteering, or other civic engagements	11 (37.9)
Employment	11 (37.9)
Sexual expression	7 (24.1)
Mental health care needs	6 (20.7)
Taking care of your home	5 (17.2)
Food	3 (10.3)
Housing	3 (10.3)
Religious or spiritual life	2 (6.9)
Physical health care needs	1 (3.4)

### Service Utilization

CAPS participants reported an average of 13.2 (SD = 9.6, Minimum = 4, Maximum = 32) meetings with a CAPS peer specialist in the first three months that they participated in the program. In a typical week, program participants reported that they met with a CAPS peer specialist for about 2.59 h (SD = 0.98, Minimum = 1, Maximum = 4). Program participants most frequently met with a CAPS peer specialist at their home (M = 9.4, SD = 7.5) followed by meetings in the community (M = 3.8, SD = 5.1) or at the CAPS program location (M = 0.5, SD = 1.3).

### Working Alliance Inventory

Among the 19 participants who responded to the WAI-S at the 3-month follow-up, the mean of the WAI-S total scores was 4.07 (SD = 0.74, ranging from 2.83 to 5.00) out of a possible 5, which corresponded to “very often” on the Likert-like scale. Similarly, high scores were also observed in the Bond, Goal, and Task subscales. The descriptive statistics for the subscales are presented in Table 3.

**Table 3:** Means and standard deviations of the Working Alliance Inventory – Short Form (WAI-S) and subscales at the 3-month follow-up (N = 19)

	M	SD
WAI-S total score	4.07	0.74
WAI-S Bond	4.39	0.68
WAI-S Goal	4.21	0.78
WAI-S Task	3.62	1.03

### Perceived Satisfaction

#### CAPS Participants

The overwhelming majority of CAPS participants (18 out of 20, or 90%) reported overall satisfaction (i.e., responded “agree” or “strongly agree” on the items) with the program (Fig. 3). Most of the participants agreed that CAPS peer specialists believed that CAPS participants could choose what was best for themselves (95%, n = 19 out of 20), that they could grow and change (84%, n = 16 out of 19) and that they took responsibility for how they lived their life (80%, n = 16 out of 20). Most CAPS participants (80%, n = 16 out of 20) agreed or strongly agreed that they would participate in the CAPS service again. CAPS participants were less enthusiastic about other aspects of their experience with the program, including 15 out of 20 (75%) who agreed or strongly agreed that CAPS were willing to see them as often as they felt necessary, 14 (70%) who indicated they would recommend the CAPS service to a friend or family member, and 13 (65%) who felt the CAPS were sensitive to their cultural or ethnic background. While their ratings were all fairly high in these areas, they fell below the desired benchmark of 80%.

#### Peer Specialists

The perceived satisfaction of peer specialists is presented in Fig. 4. Among the five peer specialists who completed this measure, at least four of five peer specialists agreed or strongly agreed with all items. Specifically, all peer specialists agreed that they and their peer specialist peers were sensitive to the cultural or ethnic background of the people they worked with; encouraged them to take responsibility for how they lived their life; believed that they could grow and change; and were willing to see them as often as they felt it were necessary. 

## Discussion

Autistic-delivered peer support is a promising new service option for autistic adolescents and adults. Using a program modeled from peer support services widely established for individuals with mental health diagnoses, the Community Autism Peer Support (CAPS) program is among the first to develop a specialized peer support training program for autistic individuals and implement an autistic-delivered peer support program. The co-development of CAPS with autistic individuals substantively enriched the program content, evaluation, and sustainability. The ability for autistic-delivered peer support to be implemented in one of the largest cities in the US with among the highest proportion of the population living in poverty underscores the versatility of and demand for service options that are developed based on the needs of the communities they intend to serve.

We provide empirical data about the feasibility and acceptability of an autistic-delivered peer support intervention for transition-age youth and adults. Data from 29 participants who were referred to the program suggest that autistic-delivered peer support services using the CAPS model are feasible for autistic adults with multiple and intensive service and support needs. Participants were generally highly engaged in the services and reported high levels of satisfaction with most aspects of the program.

### Demand

CAPS can be delivered to autistic youth and adults with complex, significant needs, including many with co-occurring mental health diagnoses and social communication needs. About half of the program participants reported at least one mental health diagnosis. Most of the adult program participants (67.8%) in our sample were in the moderate to severe deficiency range defined by the SRS. Program participants had a wide range and high number of needs in their social lives, physical activity, mental health service and supports, and daytime activities. More than half of the participants also reported autism treatment, transportation, finance, and education support needs. Future research should examine the relationship between services received and needs of program participants, as well as identify the impact of the CAPS program on community functioning that autistic individuals prioritize.

CAPS participants, again, many with complex needs, were also fairly engaged in the program. The median number of contacts was 9 and the mean was 12.9. Reasons for varied engagement need to be further studied and could reflect the continuum of needs across the autism spectrum, fluctuation in needs across the lifespan, participants’ satisfaction with the support received, or practical barriers to meeting more frequently. Given CAPS is a self-directed treatment service, variability in service use and service delivery is not uncommon. CAPS is intended to address geographical barriers by maximizing flexibility for meeting times and locations (e.g., at participants’ home, in the community). Due to the COVID-19 pandemic, CAPS contacts with participants were predominantly virtual beginning in March 2020. The modality of service delivery should be examined in the future. Research on the use of telehealth among autistic individuals has primarily been limited to children and to diagnostic and assessment services (Sutherland et al., 2018; Valentine et al., 2021). System-wide enhancements to support telehealth may dually benefit CAPS replication and expansion, including in rural areas.

### Practicality

Connections between peer specialists and participants were documented and provided initial evidence for the expansion and replication of CAPS. Positive relationships with the peer specialists, positive reports about participation, and favorable peer specialist feedback regarding the program were observed. Both program participants and peer specialists reported high overall satisfaction with the CAPS service and the people working in the program. Most peer specialists and program participants would choose to participate in this program again. It is worth noting, however, that we only have data from a few peer specialists. Future efforts should include more peer specialists to draw a conclusion about their satisfaction delivering the program. Many aspects of these connections warrant further study. There may be specific elements of matching peer specialists to participants that would warrant focused inclusion in the program structure. Peer supports are not a clinical intervention and there may be characteristics of communication styles or experiences within communities that yield an optimal fit between peer specialists and participants. Alignment of peer support with community needs or goals, for example in employment, may be ideal platforms for determining if there may be processes for assessing or assigning fit between peer specialists and participants. Further, peer support presents a service option that is responsive to the recent call for stepped care and personalized health models intervention (Lord et al., 2022) as peer support can be utilized across settings and is designed to adjust to individual needs.

### Acceptability

Most (70%) program participants would recommend this program to a friend or a family member, which is very high, but did fall below our desired benchmark (i.e., 80%). Unfortunately, due to a lack of qualitative data, we could not determine the specific reasons given by a few participants for not recommending participating in CAPS. Future research should consider a mixed-method approach to further explore potential weaknesses and strengths of the program. Responses to questions about cultural sensitivity or ethnic background were also below the desired satisfaction levels (65% of participants agreed that the support they received was culturally appropriate). The establishment of CAPS was at all phases proactive in seeking to engage diverse groups, which is a departure from most autism intervention research conducted with less heterogenous groups (DuBay et al., 2018). Despite these steps, additional attention may be needed to training content or to ongoing training and support of peer specialists to ensure participants are supported regardless of cultural background. Peer specialists must recognize and value the intersectionality of autism and gender identity (e.g., LGBTQ + population), class, race and ethnicity to fully support participants, and to build rapport and strong relationships from diverse backgrounds. While the CAPS training curriculum includes a cultural competence component, this is an area where the growing understanding of best practices in training, adult learning, and supports for peer specialists can be integrated to benefit CAPS. Consistent and recurring touchpoints to discuss cultural competence among peer specialists and providing education and learning opportunities specific to diversity and inclusion are also strategies to focus intentional effort on cultural competence.

## Implication and Future Directions

Autistic-delivered peer support has the potential to fill a service need among autistic youth and adults and create a meaningful employment opportunity for them as well. Our findings underscore one of the most empowering aspects of autistic-delivered peer support and a reflection of the evolving philosophy of supporting autistic individuals: that lived experience is a core component of transformative work.

CAPS is highly novel and includes the necessary elements to achieve approval as a Medicaid billable service across states. State regulations on licensing/certification, service authorizations, and Medicaid reimbursement rates can impact the availability of a peer support workforce (Page et al., 2020). Since Medicaid is the largest payer for behavioral health services across the lifespan (Rizzolo et al., 2013; Schott et al., 2021a, 2021b; Semansky et al., 2011a), the implications of this novel autistic-delivered reimbursable peer support program are significant for expanding service access and employment opportunities both locally, statewide and nationally within this critical insurer (Kang-Yi et al., 2016; Miller et al., 2016; Semansky et al., 2011b; Watts et al., 2020). Future expansion of these services should proceed in parallel to continuing research to understand opportunities to improve service design and delivery.

Although the CAPS shows promise among individuals across the autism spectrum, further evidence is needed, especially considering a small group of participants (n = 19) and peer specialists (n = 4) that completed measures at the 3-month timepoint. To date, CAPS has included only one participant with an intellectual disability, and it does not provide tailored services for non-speaking individuals or those who use non-traditional communication methods. In alignment with the recent call to action around including individuals with the highest support needs (Lord et al., 2022), ensuring access to peer support for individuals across the spectrum is critical for an equitable service system. Additionally, while peer-delivered service is the standard of care in mental health, it is a novel service option for autistic individuals and outreach may be needed to educate individuals, families, and providers.

## Conclusions

Our findings increase the understanding of the feasibility of the CAPS program for providing individualized services to autistic youth and adults with diverse needs. Our preliminary results suggest that CAPS can be feasible in the community setting, resulting in positive peer relationships and high satisfaction with the program with the need for future improved study design with bigger sample sizes. The autistic-delivered peer support model examined in this study was built upon collaborative stakeholder relationships that valued and prioritized autistic voices. Future studies should continue to work with stakeholders in evaluating if the program is effective at improving autistic individual outcomes and meeting their needs.

### Supplementary Information

Below is the link to the electronic supplementary material.

Supplementary file1 (DOCX 14 KB)
