# Supplementary material for: Autistic-Delivered Peer Support: A Feasibility Study
Source: J Autism Dev Disord. 2022 Nov 12;54(2):409–22. doi: 10.1007/s10803-022-05816-4 (PMC9652131; doi:10.1007/s10803-022-05816-4)
Supplement: Supplementary file 1 — Supplementary file1 (DOCX 14 KB) [file 10803_2022_5816_MOESM1_ESM.docx]

**Appendix A**

**Working Alliance Inventory Items (Dimensions)**

1. As a result of CAPS, I am clearer as to how I might be able to change. (Task)
2. What I am doing in CAPS gives me new ways of looking at my problem. (Task)
3. I believe my supervisor likes me. (Bond)
4. My supervisor and I collaborate on setting goals for my wellness. (Goal)
5. My supervisor and I respect each other. (Bond)
6. My supervisor and I are working towards mutually agreed upon goals. (Goal)
7. I feel that my supervisor appreciates me. (Bond)
8. My supervisor and I agree on what is important for me to work on. (Goal)
9. I feel my supervisor cares about me even when I do things that they do not approve of. (Bond)
10. I feel that the things I do in CAPS will help me to accomplish the changes that I want. (Task)
11. My supervisor and I have established a good understanding of the kind of changes that would be good for me. (Goal)
12. I believe the way we are working with my needs is correct. (Task)
